# A Toolbox for the Generation of Chemical Probes for Baculovirus IAP Repeat Containing Proteins

**DOI:** 10.3389/fcell.2022.886537

**Published:** 2022-05-26

**Authors:** Martin P. Schwalm, Lena M. Berger, Maximilian N. Meuter, James D. Vasta, Cesear R. Corona, Sandra Röhm, Benedict-Tilman Berger, Frederic Farges, Sebastian M. Beinert, Franziska Preuss, Viktoria Morasch, Vladimir V. Rogov, Sebastian Mathea, Krishna Saxena, Matthew B. Robers, Susanne Müller, Stefan Knapp

**Affiliations:** ^1^ Department of Biochemistry, Chemistry and Pharmacy, Institute for Pharmaceutical Chemistry, Goethe University, Frankfurt, Germany; ^2^ Structural Genomics Consortium, Buchmann Institute for Molecular Life Sciences, Goethe University, Frankfurt, Germany; ^3^ Promega Corporation, Madison, WI, United States; ^4^ German Cancer Consortium (DKTK), German Cancer Research Center (DKFZ), Heidelberg, Germany

**Keywords:** IAP, E3 Ligase, PROTAC, Ubiquitin, NanoBRET

## Abstract

E3 ligases constitute a large and diverse family of proteins that play a central role in regulating protein homeostasis by recruiting substrate proteins *via* recruitment domains to the proteasomal degradation machinery. Small molecules can either inhibit, modulate or hijack E3 function. The latter class of small molecules led to the development of selective protein degraders, such as PROTACs (PROteolysis TArgeting Chimeras), that recruit protein targets to the ubiquitin system leading to a new class of pharmacologically active drugs and to new therapeutic options. Recent efforts have focused on the E3 family of Baculovirus IAP Repeat (BIR) domains that comprise a structurally conserved but diverse 70 amino acid long protein interaction domain. In the human proteome, 16 BIR domains have been identified, among them promising drug targets such as the Inhibitors of Apoptosis (IAP) family, that typically contain three BIR domains (BIR1, BIR2, and BIR3). To date, this target area lacks assay tools that would allow comprehensive evaluation of inhibitor selectivity. As a consequence, the selectivity of current BIR domain targeting inhibitors is unknown. To this end, we developed assays that allow determination of inhibitor selectivity *in vitro* as well as *in cellulo*. Using this toolbox, we have characterized available BIR domain inhibitors. The characterized chemical starting points and selectivity data will be the basis for the generation of new chemical probes for IAP proteins with well-characterized mode of action and provide the basis for future drug discovery efforts and the development of PROTACs and molecular glues.

## Introduction

**FIGURE 1:**
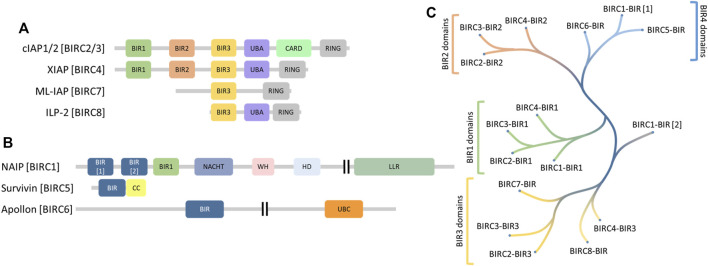
**(A)** domain structure of the BIRC family E3 ligases displaying conservation of the BIR3 domain (yellow) and the RING domain (grey). **(B)** domain structure of the residual BIRC family members showing no conservation. **(C)** phylogenetic analysis of the BIR domains from all BIRC proteins showing clustering for the BIR1 (green), BIR2 (orange) and BIR3 (yellow) domains together with some ungrouped domains (blue). BIRC, Baculovirus IAP Repeat containing; BIR, Baculovirus IAP Repeat; UBA, Ubiquitin-associated; CARD, Caspase recruitment domain; RING, really interesting new gene; CC, Coiled coil; NACHT, NAIP, CIITA, HET-E und TEP1; WH, Winged helix; HD, Helical domain; UBC, Ubiquitin-conjugating; LLR, Leucine-rich repeat.

Human inhibitors of apoptosis (IAP) proteins, also often referred to as baculoviral IAP repeat-containing proteins (BIRCs) comprise a family of proteins sharing a homologous domain, called baculoviral IAP repeat (BIR) domain. The BIRC protein family consists of five Really Interesting New Gene (RING) type E3 ligases (BIRC2-4, 7 and 8, Figure 1A) and 3 non-E3 ligases (BIRC1, BIRC5-6, Figure 1B) that lack the RING domain. BIRC2 and BIRC3, each comprise three BIR domains (BIR1-3), a ubiquitin-associated (UBA) domain, a caspase recruitment domain (CARD) and a RING domain. The UBA ubiquitin interacting domain is also present in BIRC4 and BIRC8 but not BIRC7. All BIRC E3 ligases contain in addition to the BIR3 domain a C-terminal RING domain, which are both required for their protein degradation activity (Deshaies and Joazeiro, 2009). BIRC2/3 E3 ligases are involved in the modulation of diverse pathways including TNFα regulation through TRAF2 degradation and the regulation of the NFκB pathway (Varfolomeev et al., 2007; Vince et al., 2007). Also BIRC4 has been reported to activate the NFκB pathway but plays additional roles in TGFβ signaling, mammary gland development and maturation of T-cells. Deficiency of BIRC4 in humans causes defects in immunity such as susceptibility to infections, splenomegaly, cytopaenias, and autoinflammatory disease (Mudde et al., 2021). BIRC7 acts as an activator of non-apoptotic functions of caspases such as spermatogenesis. BIRC8 was reported to be active in immune deficiency response as a result of *E. coli* infection (Dubrez-Daloz et al., 2008).

Non-E3 ligases include BIRC5, a small protein which contains only a single BIR domain with an adjacent coiled-coil domain. BIRC5 plays a role in mitosis and has also been described as a promising cancer target (Altieri, 2003; Wheatley and Altieri, 2019). In contrast, BIRC6 is a large (4857 amino acid) protein consisting of only two annotated domains thus far, a single BIR domain and a UBC domain. BIRC6 has been described to act as a ubiquitin conjugating enzyme (Ikeda, 2018) functioning as a negative regulator of autophagy (Jia and Bonifacino, 2019). The third non-E3 ligase, BIRC1, is a multidomain protein containing 3 BIR domains. While BIRC1 has been published to act as part of the inflammasome that assembles after bacterial infection (Vance, 2015), the role of its BIR domains is not fully understood.

BIRCs have been shown to inhibit apoptosis of cells, while uncontrolled BIRC activity leads to resistance of regulated cell death, an acquired property that constitutes one of the hallmarks of cancer (Hanahan, 2022). Biological roles of BIRCs have been extensively reviewed (Salvesen and Duckett, 2002; Dubrez-Daloz et al., 2008; Berthelet and Dubrez, 2013). X-linked IAP (XIAP/BIRC4) is one of the best characterized family members and is considered a target for therapeutic intervention in several cancers (Eckelman et al., 2006).

The natural inhibitor of XIAP is the so called Second Mitochondria-derived Activator of Caspases/Direct IAP Binding with Low pI (SMAC/Diablo), an N-terminal tetrapeptide (AVPI) which inspired IAP antagonists, called “SMAC mimetics” binding to the IAP binding motif (IBM) groove of some BIR domains (Salvesen and Duckett, 2002; Berthelet and Dubrez, 2013). Monovalent SMAC mimetics as well as bivalent compounds for the simultaneous binding of two BIR domains have been developed. Bivalent inhibitors may either act in *cis* in order to increase affinity towards a single BIRC protein through simultaneous binding to BIR3 and BIR2 (only applicable for BIRC2-4), or in *trans* resulting in the recruitment of two BIRC E3 ligases and ubiquitinylation followed by proteasomal degradation (Supplementary Figure S1). Monovalent SMAC mimetics may also be used for the design of specific PROTACs called SNIPERs (Specific and Non-genetic Inhibitor of apoptosis protein (IAP)-dependent Protein ERasers) which led to efficient degradation of a number of diverse target proteins (Naito et al., 2019). Despite the increasing number of SMAC mimetic inhibitors including a number of compounds in clinical evaluation, no comprehensive assay platforms have been established to characterize the selectivity of BIRC inhibitors within this family of protein interaction domains (Morrish et al., 2020).

Fluorescence polarization has been widely used for the characterization of BIR domain inhibitors (Nikolovska-Coleska et al., 2004), but particular cellular target engagement assays covering full length as well as single BIR domains such as NanoBRET (Nano Bioluminescence Resonance Energy Transfer) would represent a versatile platform ideally suited to assess domain selectivity and on-target inhibitor engagement in cells (Vasta et al., 2018). In live-cell NanoBRET assay, the protein of interest is usually (transiently) expressed in mammalian cells, enabling inhibitor studies with full-length proteins as well as single targeted domains in the cellular environment. Thus, binding studies of an inhibitor to the full-length protein expressed with appropriate post-translational modifications are possible in this assay format. In addition, kinetic studies, providing information of target residence time are possible as well using inhibitor wash-out experiments. Here, we report the development and utilization of a cell-based BIRC protein family selectivity platform based on NanoBRET technology as a toolbox for the development of BIR domain targeting inhibitors, optimization of PROTACs and the characterization of on-target activity of chemical BIR domain probes.

## Materials and Methods

### Inhibitors

SM-164 and GDC-0152 were purchased from Cayman Chemical (#28632 and #17810). AZD5582, BV-6, Birinapant, LCL161 and AT406 were purchased from Selleckem (#S7362, #S7597, #S7015, #S7009, and #S2754). A 410099.1 and UC 112 were purchased from Tocris Bioscience (#6470 and #5251). In addition, CUDC-427 was purchased from MedChemExpress (#HY-15835). Quality control of the purchased compounds was carried out by HPLC-MS mass validation which confirmed the expected molecular weight of all purchased inhibitors.

### Phylogenetic Analysis of the BIRC Protein Family BIR Domains

For phylogenetic analysis of the BIR domains, protein sequences were obtained from UniProt and aligned using the MAFFT sequence alignment tool available in the “MPI Bioinformatics Toolkit” (Gabler et al., 2020). The obtained multiple sequence alignment was used as input an file for iTOL (Letunic and Bork, 2021) for the phylogenetic analysis and generation of a newick file for further analysis in MEGA (Kumar et al., 2018). The obtained multiple sequence alignment of all human BIR domains is shown in Supplementary Figure S2.

### Tracer Synthesis

IAP amine (Ohoka et al., 2017) (25.0 mg, 0.034 mmol) was charged into a 100 ml round bottom flask and was dissolved in DMF (5.0 ml) by stirring. The resulting solution was treated with the Hünig base N,N-Diisopropylethylamine (DIPEA) (29.3 µL, 0.168 mmol) and allowed to stir for 10 min. To the stirred mixture the BODIPY dye NHS (N-Hydroxysuccinimide) ester TM 590 SE (14.3 mg, 0.034 mmol) was added and the reaction was allowed to stir in a capped glass vessel in the dark for 3 h. The mixture was diluted with 1:1:0.01 water, acetonitrile, trifluoroacetic acid (TFA) (4 ml) and was subjected to reverse-phase preparative HPLC purification using a 30 min linear gradient of 0.1% TFA/water (10%) in acetonitrile (90%) to 100% acetonitrile. Product containing fractions were analyzed by LCMS to confirm product identity and purity and pure fractions were pooled. Volatiles were removed under reduced pressure to give a purple film that was re-dissolved with 10 ml acetonitrile and concentrated to dryness x 3. The resulting purple residue (16.5 mg, 44.0%) was dissolved in DMSO. MS(ESI) was measured. The m/z was compared with the calculated mass for the expected molecule C_48_H_60_BF_2_N_8_O_7_S (M + H) 941.44 Da with the found mass of 941.5 Da confirming the structure of the synthesized BRET tracer.

### Protein Expression and Purification

BIRC2-BIR3 domain (260–352) was expressed as a recombinant fusion protein incorporating a His_6_ and GST tag at the N-terminus. *E. coli* were cultured in Terrific Broth (TB) at 37°C until an OD_600_ of 1.0 was reached. The culture was then cooled to 18°C and allowed to reach an OD_600_ of 2.5. Protein expression was induced by the addition of 0.5 mM isopropyl β-D-1-thiogalactopyranoside (IPTG) and the protein was allowed to express overnight. Cells were harvested (Beckman centrifuge, via centrifugation at 6000 g at 4°C) and lysed by sonication (SONICS vibra cell, 5 s on-, 10 s off cycle using a total of 30 min) in the presence of DNase I (Roche, Basel, CH) and cOmplete EDTA-free protease inhibitor (Roche, Basel, CH), and recombinant protein was purified using GST-affinity chromatography in Purification buffer [30 mM 4-(2-hydroxyethyl)-1-piperazineethanesulfonic acid; pH 7.5 (HEPES), 500 mM NaCl, 5% glycerol, 0.5 mM tris(2-carboxyethyl)phosphine (TCEP)] and elution was carried out using Purification buffer including additional 10 mM reduced glutathione. The eluted proteins were dialysed overnight into gel filtration buffer (30 mM HEPES pH 7.5, 250 mM NaCl, 5% glycerol and 0.5 mM TCEP) while the expression tag was cleaved using 1 mg tobacco etch virus (TEV) protease. The cleaved protein was passed through a HiLoad^®^ 26/600 Superdex^®^ 75 pg (GE Healthcare) size exclusion chromatography column and the resulting pure protein was stored in gel filtration buffer, flash frozen in liquid nitrogen and subsequently stored at −80°C for further experiments.

### Isothermal Titration Calorimetry

ITC experiments were performed using a NanoITC instrument (TA Instruments, New Castle, United States) at 25°C in gel filtration buffer (30 mM HEPES pH 7.5, 250 mM NaCl, 5% glycerol and 0.5 mM TCEP). Purified BIRC2-BIR3 protein at a concentration of 116 μM was titrated into the reaction cell containing 10 µM inhibitor dissolved in gel filtration buffer. For this protocol, the chamber was pre-equilibrated with the test compound, and the BIRC2-BIR3 domain was titrated in while continuously measuring the rate of exothermic heat evolution. The heat of binding was integrated, corrected, and fitted to an independent single-binding site model based on the manufacturer’s instructions, from which thermodynamic parameters (ΔH and TΔS), equilibrium association and dissociation constants (*K*_A_ and *K*_D_, respectively), and stoichiometry (n) were calculated. Measurements were carried out in technical duplicates except for the inhibitor UC-112. Data were displayed using GraphPad Prism 9.3.

### Differential Scanning Fluorimetry Assay

Differences in the melting temperature (Δ*T*_m_) data were measured as described in Fedorov et al. (2012). Purified proteins were buffered in DSF buffer (25 mM HEPES pH 7.5, 500 mM NaCl) and were assayed in a 384-well plate (Thermo, #BC3384) with a final protein concentration of 20 μM in 10 μL final assay volume. Inhibitors were added in excess to a final concentration of 40 μM, using an ECHO 550 acoustic dispenser (Labcyte). As a fluorescent probe, SYPRO-Orange (Molecular Probes) was used at 5x final concentration. Filters for excitation and emission were set to 465 and 590 nm, respectively. The temperature was increased from 25°C with 3°C/min to a final temperature of 99°C, while scanning, using the QuantStudio5 (Applied Biosystems). Data was analyzed using Boltzmann-equation in the Protein Thermal Shift software (Applied Biosystems). Samples were measured in technical triplicates.

### Fluorescence Polarization Assay

For the complementation assay, the fluorescently labeled SMAC probe (AVPIAQKSEK-K(5-FAM)-NH2) was diluted to 2-fold *K*_D_ (30 nM) in assay buffer (50 mM HEPES pH 7.5, 150 mM NaCl, 5% glycerol, 1 mM TCEP and 0.05% TWEEN20) in a black 384-well flat bottom plate (Greiner Bio-One, #784076) and purified BIRC2-BIR3 domain was titrated in a concentration range from 40 μM to 600 pM. After 1 h incubation at room temperature, fluorescence polarization was measured with excitation wavelength of 485 nm and emission wavelength of 535 nm, respectively, using a Tecan Spark plate reader (TECAN). Resulting data was plotted using GraphPad Prism 9.3 software and analyzed using a nonlinear fit to calculate the probe IC_50_. For competition assays, 5 nM probe was added to assay buffer containing 30 nM BIRC2-BIR3 domain (2x IC_50_). Compounds were titrated from 20 μM to 20 nM using an ECHO 550 acoustic dispenser (Labcyte) incubated for 1 h at room temperature and subsequent read out. Data was plotted in GraphPad Prism 9.3 and analyzed using a nonlinear fit [equation: Y = 100/(1 + 10^((X-LogIC50)))] for IC_50_ determination. *K*_I_ calculation was performed using the Cheng-Prusoff equation (Yung-Chi and Prusoff, 1973) [*K*_I_ = IC_50_/(1+([*R*]/*K*_D_))] with *K*_D_ = 15 nM, [*R*] = 30 nM and IC_50_ determined in each assay.

### NanoBRET Cellular Target Engagement Assay

The assay was performed as described previously (Vasta et al., 2018). In brief: Constructs contained the cDNA of full-length or single domains cloned in frame with an N-terminal NanoLuc-fusion as specified in Supplementary Table S2. Plasmids were transfected into HEK293T cells using FuGENE HD (Promega, E2312) and proteins were allowed to express for 20 h. Serially diluted inhibitor and NanoBRET IAP Tracer (Promega) at a concentration determined previously as the IAP Tracer K_D, app_ (Supplementary Table S2) were pipetted into white 384-well plates (Greiner 781 207) using an Echo 550 acoustic dispenser (Labcyte). The corresponding transfected cells were added and reseeded at a density of 2.5^5^ cells/mL after trypsinization and resuspending in Opti-MEM without phenol red (Life Technologies). The system was allowed to equilibrate for 2 h at 37°C and 5% CO_2_ prior to BRET measurements. To measure BRET, NanoBRET NanoGlo Substrate + Extracellular NanoLuc Inhibitor (Promega, N2540) was added as per the manufacturer’s protocol, and filtered luminescence was measured on a PHERAstar plate reader (BMG Labtech) equipped with a luminescence filter pair [450 nm BP filter (donor) and 610 nm LP filter (acceptor)]. Competitive displacement data were then graphed using GraphPad Prism 9.3 software using a normalized 3-parameter curve fit with the following equation: Y = 100/(1 + 10^(X-LogIC_50_)).

## Results

### Phylogenetic Analysis of Human BIR Domains

The human proteome encodes for 16 BIR domains that are present in 8 different BIRC proteins. The first objective towards a selectivity platform was to analyze the similarity of the different BIR domains and we therefore aligned the individual BIR domains based on their amino acid sequence homology. The resulting tree is depicted in Figure 1C. The tree revealed that the 16 BIR domains can be grouped into four major groups based on sequence homology. In the past, BIR domains were numbered starting from their N- to their C-terminus, irrespective of their sequence homology. The established BIR domain family tree revealed the three canonical groups which matched the established categories of BIR1-3 while the N-terminal BIR domain in BIRC1 (BIRC1-BIR[1]), and the unique BIR domains in BIRC5 and BIRC6 clustered into an independent family. Interestingly, the third BIR domain of BIRC1 was found to cluster with BIR1 domains of BIRC2-4 and we propose to rename this domain as BIRC1-BIR1, whereas the first BIR1 domain showed sequence homology with BIRC5 and BIRC6, supporting the existence of an additional group of BIR domains, which we named “BIR4” domains and which we have labelled BIRC1-BIR[1]. The second BIR domain of BIRC1, BIRC1-BIR[2], did not cluster with other BIR2 domains and it was located between BIR2 and BIR3 branches due to only weak sequence homology with other BIR domains.

### Biophysical Characterization of the Interaction of BIRC Literature Compounds to Single BIR Domains

Diverse *in vitro* biophysical assays including fluorescence polarization (FP) assay, differential scanning fluorimetry (DSF) and isothermal titration calorimetry (ITC) were set up for initial characterization of SMAC inhibitor binding. Since thus far most medicinal chemistry approaches have targeted the BIR3 domain of BIRC2 (BIRC2-BIR3), this domain was chosen as a representative BIR domain for a comprehensive analysis. Each of the chosen assays complements the other in its ability to characterize the binding of a chemical compound to the BIR domain. Using these assays, 10 diverse and commercially available inhibitors were selected (Supplementary Figure S1). The compounds included both monovalent as well as bivalent inhibitors with described *K*_I_ values from the literature ranging from 0.3 nM for SM-164 (Lu et al., 2008) to 169 nM for BV-6 towards the BIR domains (Varfolomeev et al., 2007).

Fluorescence polarization (FP) assays have often been the first choice for *in vitro* BIRC compound characterization. The assay is based on the displacement of a fluorescent SMAC mimetic from the binding site. The first step for establishment of the assay was the *K*_D_ determination of the tracer to the BIRC2-BIR3 domain, which was determined to be 14.9 nM (Supplementary Figure S3A). Overall, most of the compounds showed potent displacement of the tracer and tight binding to the BIRC2-BIR3 domain.

**FIGURE 2:**
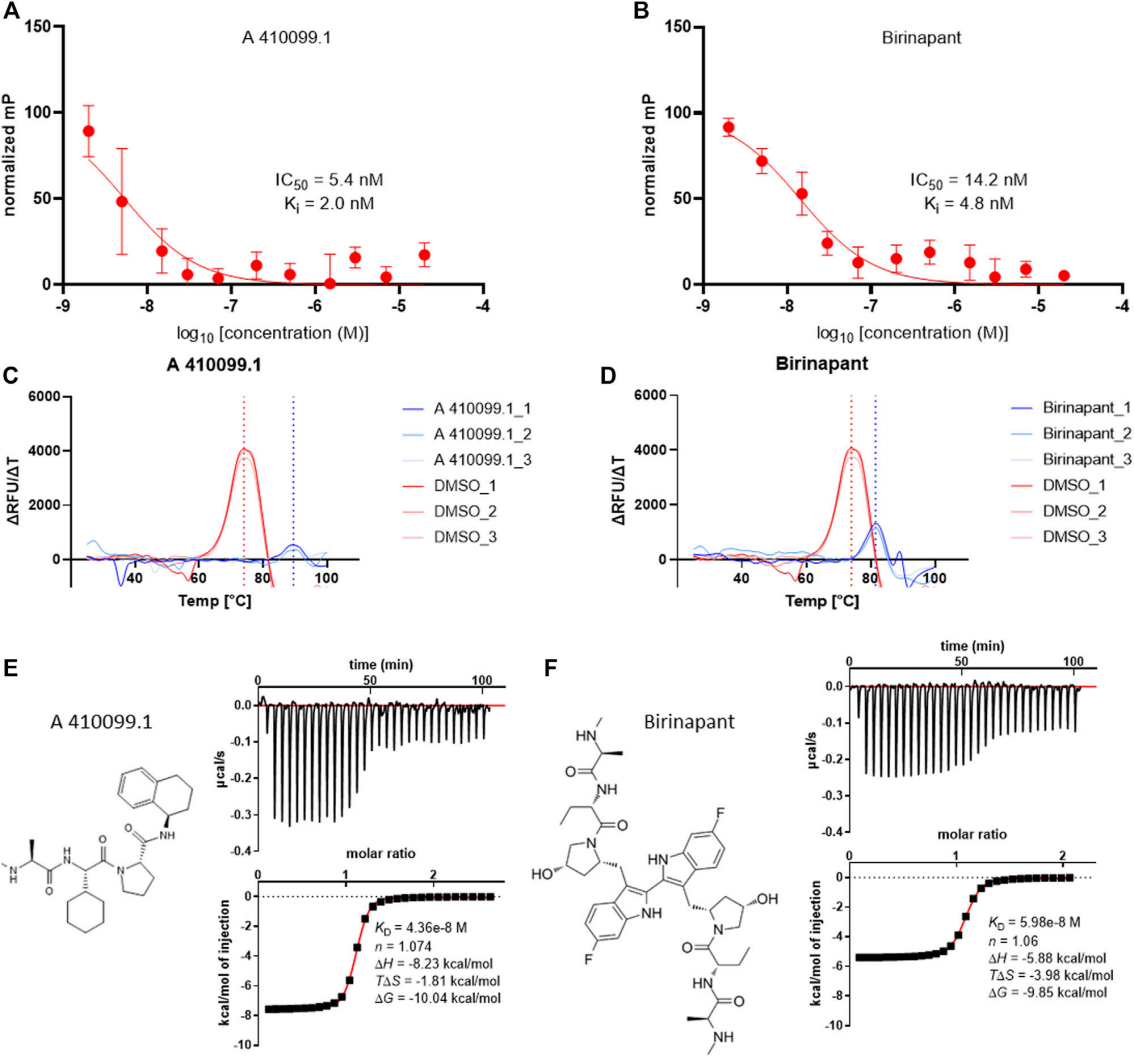
Exemplary results of the biophysical assays performed on the BIR3 domain of BIRC2. **(A)** and **(B)** FP assay curves of the compound titrations Data were expressed as mean ± SD (*n* = 3). **(A)** titration curve of A 410099.1 with an estimated IC_50_ of 5.4 nM and the calculated *K*_I_ of 2.0 nM. **(B)** titration curve of Birinapant with an estimated IC_50_ of 14.2 nM and the calculated *K*_I_ of 4.8 nM. **(C)** and **(D)** ΔRFU/ΔT plotted against the temperature obtained from DSF (*n* = 3). **(C)** melting point determination for A 410099.1 (blue dotted line) in comparison to the DMSO control (red dotted line). **(D)** melting point determination for Birinapant treated protein in comparison to the DMSO control. **(E,F)** ITC results of A 410099.1 and Birinapant with µcal/s plotted against the time in the upper frame and kcal/mol of injection plotted against the molar ratio in the bottom frame with a *K*_D_, n, ΔH, TΔS and ΔG given (*n* = 2).

Nine of the 10 tested compounds showed potent IC_50_ values between 1.8 ± 0.7 nM (SM-164) and 8.4 ± 0.7 nM (BV-6) with no discernable difference between monovalent and bivalent compounds derived from the same SMAC mimetic inhibitor. As the nonlinear fits did not reach their respective lower plateaus due to the high affinity of the binders, below the protein concentration used in the assay, these numbers represent approximations (Figures 2A,B and Supplementary Figure S3B). UC-112 binding could not be shown towards the tested BIRC2-BIR3 construct (Supplementary Figure S3). Comparison to affinity values described in the literature, showed overall good agreement with reported potencies in the medium to low nanomolar range (Table 1). The only exception was BV-6 which has previously been shown to have a *K*_I_ of 169 nM, which is significantly higher than the *K*_I_ determined by us. However, for several of the tested compounds full characterization data was not available, making our comparison incomplete. We therefore decided to apply two additional biophysical assays on BIRC2-BIR3 in order to obtain a more complete set of affinity values.

**TABLE 1:** Results of the compound titrations using the fluorescence polarization assay in comparison to published values from the literature, DSF and ITC measurements.

Tested Compound	FP Assay *K*_I_ Values [nM]	Literature FP Assay *K*_I_ Values [nM]	DSF Assay ΔT_m_ [°C]	*K*_D_ Measured in ITC [nM]
BV-6	8.4 ± 0.7	169.0^1^	9.1 ± 0.0	24.8 ± 8.5
SM-164	1.8 ± 0.7	0.3 ± 0.1^2^	13.4 ± 0.2	41.6 ± 3.4
CUDC-427	2.9 ± 0.3	No FP/biophysical data available	10.8 ± 0.1	44.7 ± 11.8
UC-112	—	No FP/biophysical data available	0.3 ± 0.1	—
AT406	2.9 ± 0.6	1.9 ± 0.2^3^	11.6 ± 0.2	36.0 ± 0.5
Birinapant	4.8 ± 0.9	<1 nM^4^	7.2 ± 0.3	57.0 ± 2.8
AZD5582	2.3 ± 0.3	6.4 ± 4.3^5^ calculated from IC_50_	18.0 ± 0.1	41.1 ± 2.9
GDC-0152	3.5 ± 1.0	17.0^6^	10.6 ± 0.7	28.1 ± 0.3
LCL161	4.1 ± 0.8	No FP/biophysical data available	10.2 ± 0.3	21.1 ± 10.0
A 410099.1	2.0 ± 1.1	No FP/biophysical data available	15.7 ± 0.1	37.5 ± 6.1

The data, generated in this work was performed on BIRC2 BIR3 domain shown with its respective SD (n = 3). DSF results are specified as the difference in melting temperature (°C) to the DMSO control (n = 3). ITC measurements were run in duplicates (except for UC-112) and the calculated KD values are given in nM together with the SD.

1 Varfolomeev et al. (2007).

2 Lu et al. (2008).

3 Cai et al. (2011).

4 Allensworth et al. (2013).

5 Hennessy et al. (2013).

6 Flygare et al. (2012).

We employed an orthogonal assay which did not rely on specific tracer binding to the domains and would therefore also detect compound interactions outside the IBM grove. Differential scanning fluorimetry (DSF) was performed on the BIR3 domain of BIRC2. First, we determined the melting temperature of the native BIR3 domain of BIRC2, which had a mid-point of the unfolding transition at ∼74°C. The assay was run in the presence of DMSO to exclude DMSO influences towards melting temperature differences when testing small molecules (Figures 2C,D). Despite this high melting temperature, it was possible to further stabilize the protein up to 18°C (using AZD5582) resulting in a melting temperature of 92°C. Since the assay was conducted up to 99°C, the resulting melting curves were complete but they were at the upper limit of detection. As AZD5582 bound in the FP assay in the low nanomolar *K*_D_ range, the range of 18 °C provided a good assay window, indicating that DSF assay may be suitable for low affinity binders. Overall, 7 of the 10 compounds displayed a noticeable correlation to the FP assay, demonstrating higher shifts when higher affinities were determined in the FP assay while an exact correlation cannot be expected due to the different assay principles. The two bivalent compounds Birinapant and BV-6 were the only compounds that showed a thermal shift of less than 10°C despite potent affinities of 4.8 and 8.4 nM, respectively in the FP assay. Additionally, in agreement with our FP assay data, UC-112 did not lead to an increase in stability suggesting no binding to the studied BIR3 (Supplementary Figure S4).

As a third biophysical method for characterization of the literature compounds, isothermal titration calorimetry was performed. This method allowed the determination of accurate binding constants in solution without the need of tracer or dye molecules and additionally provide insight into the thermodynamic properties of the inhibitor interaction and the binding stoichiometry (Figures 2E,F). Generally, ITC showed dissociation constants between 20 and 60 nM for the mono- and bivalent SMAC mimetics (Table 1). In ITC, a compound such as AZD5582 which has shown an extraordinary high thermal shift in DSF showed a lower affinity than GDC-0152 suggesting that DSF data may be somewhat influenced by inhibitor chemical composition and binding mode. Binding was strongly favored by enthalpy changes between −4 and −6 kcal/mol except for BV6 which showed a large favorable binding entropy change (TΔS: 6.94 ± 0.1 kcal/mol) probably due to water displacement from the BIR domain binding site and the ligand.

### Development of NanoBRET Assays for the BIRC Family

As a critical input for the family-wide assessment of compounds in a cellular environment, we established NanoBRET assays for full-length BIRC proteins as well as individual BIR domains. All constructs were cloned using a NanoBRET-vector harboring an N-terminal NanoLuc. Unfortunately, BIRC1 and BIRC6 constructs were not obtained. BIRC1 full-length cloning failed, resulting in accumulation of mutations when transformed into *E. coli*. Individual BIRC1-BIR domains however were successfully obtained, indicating potential toxicity of the full-length BIRC1 towards *E. coli* despite using a T7/CMV promoter which should not be transcribed by the used strain. We were not able to obtain a BIRC6 NanoLuc expression construct due to the size of over 14500 nucleotides and the lack of a suitable cDNA template. BIRC5 full-length protein only contains an additional helix in addition to the BIR domain and the full-length sequence was therefore considered as a single BIR domain construct.

**FIGURE 3:**
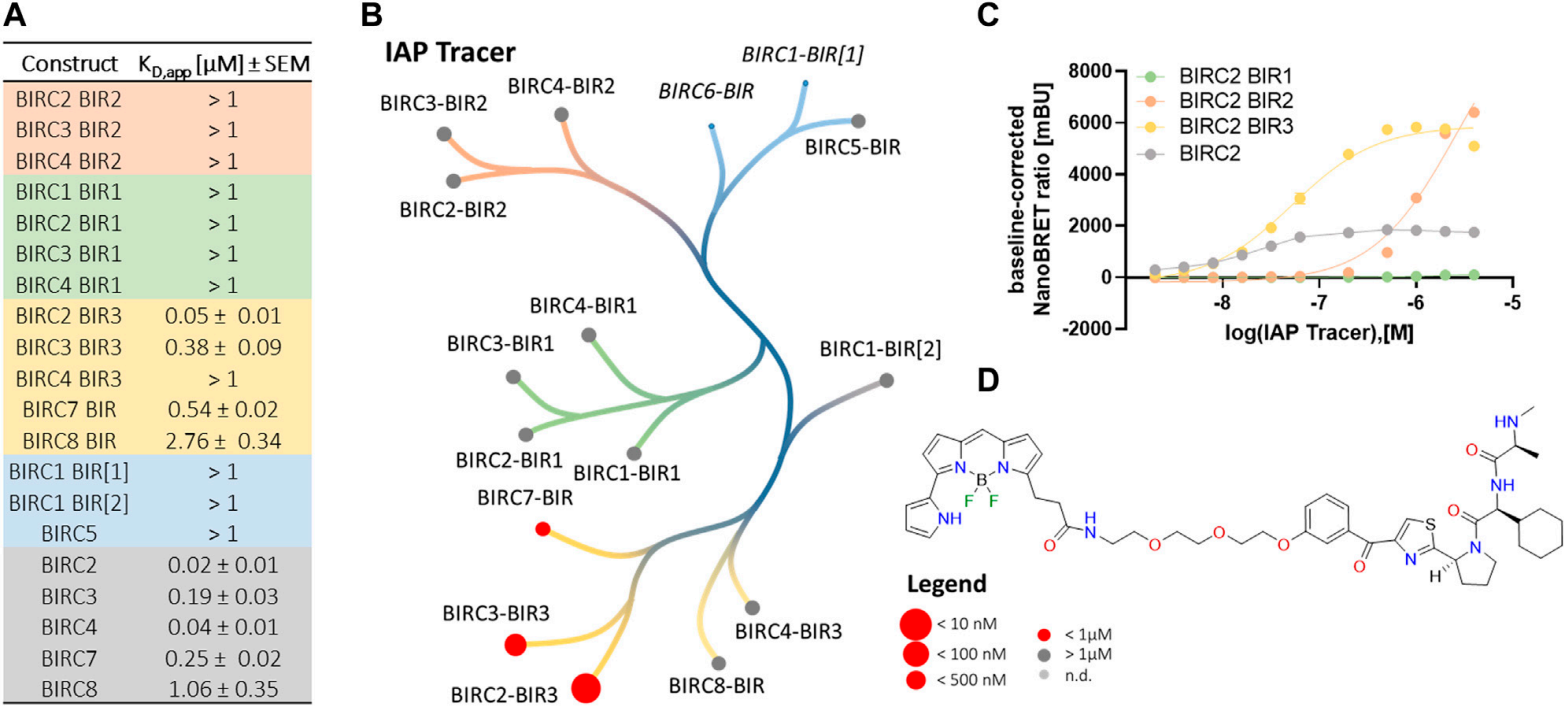
NanoBRET tracer titrations of the different BIRC constructs. **(A)** Determination of the binding affinities of the tracers to the different BIRC constructs. Data were expressed as mean ± SEM using two independent experiments performed in duplicates (*n* = 4). **(B)** Tracer potency across the BIRC family. Here, BIRC6 was not tested and the BIRC1-BIR1 domain did not show a significant luciferase signal and therefore, both constructs were excluded (italic). **(C)** Tracer titration curves for BIRC2 full-length proteins and its corresponding BIR domains (*n* = 4). **(D)** Structure of the NanoBRET IAP tracer.

Since the development of SMAC mimetics has been based on the AVPI motif of SMAC, all compounds were expected to bind to the IBM groove. Based on this, a pan-BIRC tracer was synthesized, using LCL161 as parent compound (Figure 3D), which has been shown to be a potent BIRC2-BIR3 binder with a *K*_D_ of 21.1 ± 10 nM measured by ITC. The tracer was not only tested on BIR domains expected to bind SMAC mimetics, but all successfully cloned and expressed BIR domains.

We first performed titrations using the full-length BIRC proteins (Table 2). All five full-length BIRC E3 ligases bound the tracer with high affinity (Figure 3A). For BIRC2, 3, 4 and 7, a tracer *K*_D_ of lower than 250 nM was measured with z’ values of 0.6–0.7, indicating good assay quality. BIRC8 had lower affinity for the tracer. Nevertheless, a surprisingly high assay quality with a z’ of 0.8 and an assay window of 42.9 was obtained, resulting in a high-quality assay panel of full-length BIRC proteins (Supplementary Table S2).

**TABLE 2:** Compound EC_50_s ± SEM in the cellular target engagement NanoBRET assay.

Tested Compound	BIRC2	BIRC3	BIRC4	BIRC7	BIRC8
BV-6 [µM]	0.5 ± 0.1	0.8 ± 0.1	0.9 ± 0.1	2.6 ± 0.8	2.6 ± 0.2
SM-164 [nM]	5.8 ± 0.6	10.9 ± 2.4	9.9 ± 1.1	16.5 ± 6.0	85.7 ± 4.7
CUDC-427 [nM]	622.3 ± 232.1	738.9 ± 108.4	190.2 ± 26.9	38.8 ± 2.5	218.1 ± 48.3
UC-112 [µM]	>50.0	>50.0	>50.0	>50.0	>50.0
AT406 [nM]	21.7 ± 5.5	62.0 ± 8.5	28.5 ± 5.2	25.1 ± 5.2	69.3 ± 21.1
Birinapant [µM]	1.0 ± 0.2	0.8 ± 0.4	0.2 ± 0.1	0.3 ± 0.2	2.2 ± 1.1
AZD5582 [nM]	191.8 ± 99.3	81.1 ± 14.8	79.6 ± 37.5	8.0 ± 0.3	76.3 ± 0.1
GDC-0152 [nM]	9.0 ± 0.8	23.4 ± 11.0	15.3 ± 5.0	35.5 ± 9.4	133.8 ± 8.4
LCL161 [nM]	7.5 ± 1.3	25.3 ± 1.3	18.2 ± 4.2	26.4 ± 5.5	199.4 ± 21.4
A 410099.1 [nM]	4.6 ± 0.2	9.2 ± 1.0	15.6 ± 7.3	19.9 ± 2.6	93.9 ± 9.3

The assays were performed on full-length proteins BIRC2, BIRC3, BIRC4, BIRC7, and BIRC8 in two independent experiments in technical duplicates (*n* = 4).

Next, single BIR domain constructs were tested for tracer binding. Interestingly, only BIRC2, BIRC3 and BIRC7 BIR3 domains had *K*_D, app_ values lower than 1 µM. The remaining BIR domains showed *K*_D, app_ values higher than 1 µM up to 4 μM, a range, where concentration effects of the tracer may lead to false BRET signals. The tracer affinity towards the BIRC2-BIR3 domain was shown to be 50 ± 10 nM which indicated that no significant steric problems caused by the attachment of the fluorophore occurred. Despite the lower affinity to the single domains, the assay properties were not significantly affected. For the majority of the single BIR domains, z’ values higher than 0.5 were achieved together with assay windows larger than 15. For the BIR2 and BIR3 domains, the assay windows generally were shown to be around 3-fold higher compared to the full-length protein. BIR2 domains and BIR1 domains, however showed negative z’ values indicating unsuitable assay properties (Supplementary Table S2). Exemplary tracer titration curves for all BIRC2 constructs are shown in Figure 3C.

The determined affinities of the tracer towards the BIR domains and full-length constructs were used for the subsequent compound titrations using the tracer concentration at its *K*_D, app_ in order to have comparable tracer competition according to Cheng-Prusoff (Yung-Chi and Prusoff, 1973) among the tested BIRC proteins. Domains yielding *K*_D,app_ > 1 µM were incubated with 1 µM tracer to avoid solubility problems linked to the tracer fluorophore. Since the tracer is based on the literature compound LCL161 (Figure 3D), the data measured on the BIR3 domain of BIRC2 were compared to the *in vitro* biophysical characterization we performed. The tracer showed a *K*_D, app_ of 50 ± 10 nM on the BIRC2-BIR3 domain (Figure 3A) and therefore compares well to the biophysical data collected by ITC while taking into account that cells not only have a membrane as penetration barrier but also competitive binding from endogenous proteins. Unexpectedly, assays using the full-length protein showed slightly higher affinity of 20 ± 10 nM. Here, a possible explanation could be that the environment in the context of full-length proteins favorably influences binding or that the full-length proteins show higher stability in comparison to the truncated proteins. Similar behavior of full-length compared to results for individual BIR domains was observed for the other BIRC family proteins including the full-length proteins containing only a single BIR domain which are known to form dimers (Figures 3A,B). This phenomenon was observed for all single domains in comparison to full-length except for BIRC4, where only the full-length construct resulted in a stable assay. Unfortunately, the BIRC1-BIR[1] luciferase signal was not sufficient for BRET measurement and tracer binding could therefore not be determined.

### Family-Wide Screening of Literature Compounds Targeting BIRC Proteins in Living Cells

**FIGURE 4:**
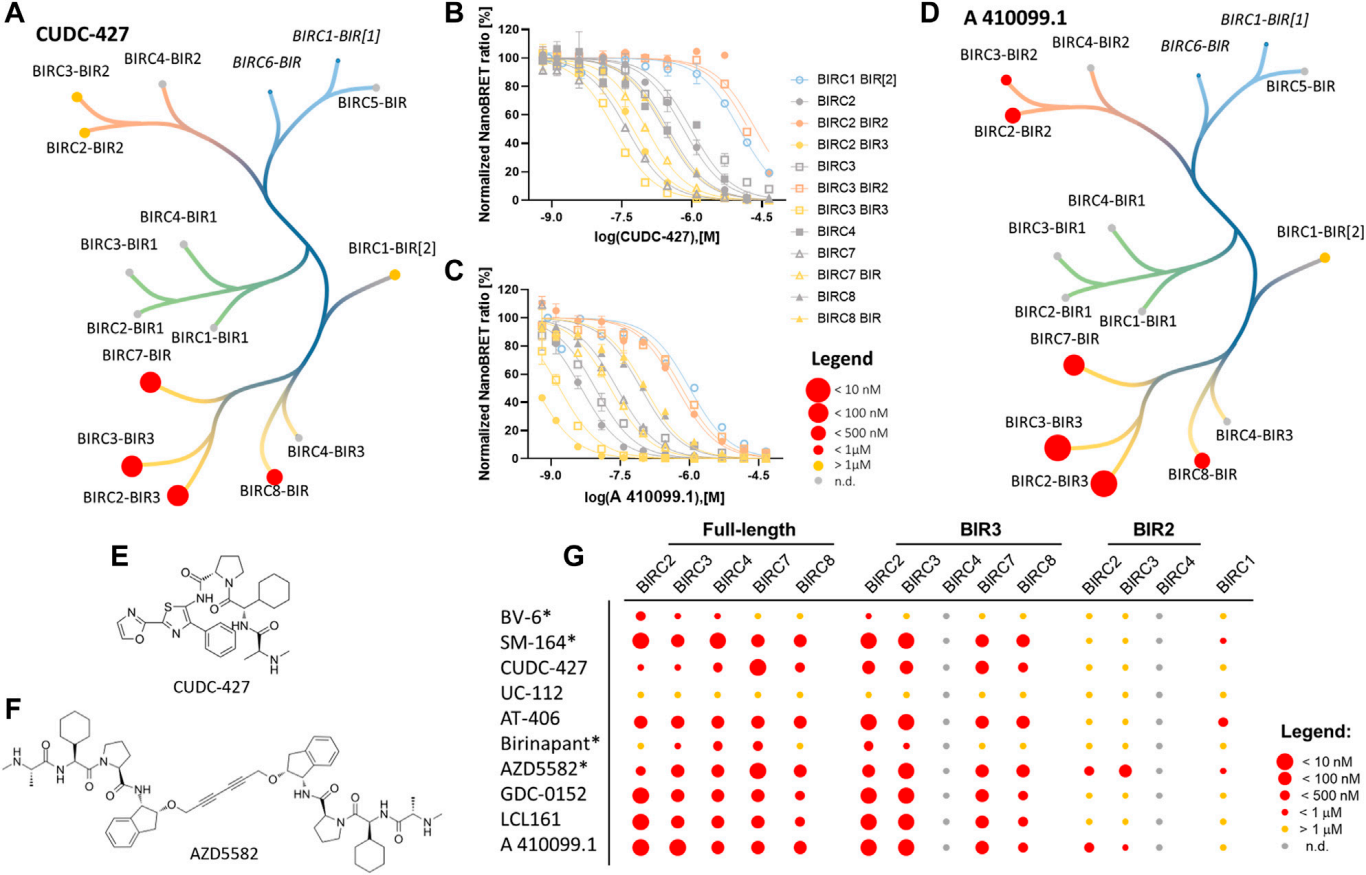
NanoBRET cellular target engagement assay using the full-length and single domain proteins. Full-length BIRC2, BIRC3, BIRC4, BIRC7, BRIC8, and BIRC1-BIR2, BIRC2-BIR2, BIRC2-BIR3, BIRC3-BIR2, BIRC3-BIR3, BIRC7-BIR and BIRC8-BIR were investigated. Data were expressed as mean ± SEM using two independent experiments performed in duplicates (*n* = 4). **(A)** Potency of CUDC-427 against tested BIRC domains. The excluded assays for BIRC6-BIR and BIRC1-BIR1 are shown in italic. **(B,C)** Normalized NanoBRET ratio [%] against the tested concentration of CUDC-427 and A 410099.1, respectively. **(D)** Potency of most potent compound A 410099.1 against tested BIRC domains. The excluded assays for BIRC6-BIR and BIRC1-BIR1 are expressed in italic. **(E,F)** chemical structures of CUDC-427 and AZD5582 which mark starting points towards BIRC7 selective inhibitors. **(G)** Heat map for compound potencies towards the corresponding protein constructs. Bivalent compounds are marked (*).

After determining the affinity of the tracer towards the different BIRC constructs and the assay quality, compound titrations were performed using the literature compounds compiled in Supplementary Figure S1. Exemplary dose-response curves obtained for each of the compound titrations are shown in Figure 4 while all dose response curves are shown in Supplementary Figure S7. We first tested the selected inhibitors using the full-length constructs. In agreement with biophysical bench marking assays, UC-112 did not bind to the full-length BIRC E3 ligases in NanoBRET assays (Table 2). All other compounds showed potent binding to all BIRC proteins.

From the bivalent compounds, BV-6 and Birinapant were the least potent inhibitors with cellular on-target affinity in the single digit µM range. Additionally, these compounds showed a significant loss in affinity in our cellular assay compared to the measured *in vitro* potency. Since SM-164 and AZD5582 did not show such a drastic loss in potency, the high molecular weight and the size of bivalent compounds cannot be the only factor influencing lower cellular activity. The monovalent compounds were highly potent in the cellular context towards the full-length proteins, indicating good membrane permeability. Most compounds were not selective for a specific BIRC protein. Interestingly, CUDC-427 (Figure 4A) was the most selective inhibitor with a cellular affinity of 38.8 ± 2.5 nM for BIRC7 but a much weaker affinity towards the remaining measured BIRC proteins ranging from 190—720 nM. Similar binding behavior was observed for AZD5582 (Table 2), indicating that selectivity for this target might be achievable. In addition to the full-length E3 ligases, the established assays for single domains were carried out (Table 3). The results of the selectivity screening were visualized on the established phylogenetic tree (Figure 4).

**TABLE 3:** Compound EC_50_s ± SEM in the cellular target engagement NanoBRET assay.

Tested Compound	BIRC2 BIR2	BIRC3 BIR2	BIRC2 BIR3	BIRC3 BIR3	BIRC7 BIR	BIRC8 BIR	BIRC1 BIR[2]
BV-6 [µM]	>50	18.0 ± 25.2	0.8 ± 0.1	1.7 ± 0.4	2.7 ± 0.8	3.0 ± 0.1	10.9 ± 2.1
SM-164 [nM]	6614.3 ± 5281.5	3467.0 ± 101.8	0.8 ± 0.1	2.3 ± 0.3	50.9 ± 44.4	87.3 ± 9.4	941.4 ± 258.3
CUDC-427 [nM]	15354.3 ± 8973.8	14840.0 ± 636.4	54.4 ± 1.0	20.0 ± 3.1	74.5 ± 40.9	271.8 ± 6.1	10120.0 ± 1230.4
UC-112 [µM]	>50	>50	>50	>50	>50	>50	>50
AT406 [nM]	7769.0 ± 1232.7	8135.0 ± 52.3	2.8 ± 0.5	5.2 ± 1.2	18.0 ± 4.5	69.6 ± 0.3	467.2 ± 21.0
Birinapant [µM]	16.6 ± 2.1	17.4 ± 1.7	0.3 ± 0.0	0.9 ± 0.0	2.3 ± 1.2	3.5 ± 0.1	18.3 ± 1.1
AZD5582 [nM]	170.6 ± 151.3	28.5 ± 3.2	23.0 ± 14.4	4.1 ± 0.1	16.1 ± 6.3	85.5 ± 11.1	775.5 ± 200.4
GDC-0152 [nM]	8856.3 ± 2312.1	12055.0 ± 502.0	1.0 ± 0.3	6.7 ± 0.4	28.4 ± 2.7	145.7 ± 3.8	15180.0 ± 1230.4
LCL161 [nM]	1752.0 ± 373.1	3183.0 ± 62.2	0.9 ± 0.2	4.7 ± 0.2	28.5 ± 7.0	258.5 ± 42.4	1486.0 ± 297.0
A 410099.1 [nM]	332.9 ± 35.4	680.5 ± 68.0	0.4 ± 0.2	1.5 ± 0.2	17.0 ± 2.4	108.1 ± 7.5	1228.2 ± 325.1

The assays were performed on single BIR domains of BIRC2, BIRC3, BIRC7 and BIRC8 and BIRC1 in two independent experiments in technical duplicates (*n* = 4).

For the single BIR domains, larger differences in binding affinities were observed. For example, BV-6 did not show any binding to BIRC2-BIR2, but potently bound to other closely related BIR2 domains. Since the compounds available in the literature were designed based on SMAC mimetics and often tested on BIRC2-BIR3, low selectivity was expected within the BIR3 domain group. Some molecules, such as the bivalent inhibitor AZD5582 bound with almost equal affinity to BIR2 as well as BIR3 domains, whereas others, such as CUDC-427 or LCL161 almost exclusively bound to the BIR3 branch of BIRC proteins (Figure 4B and Supplementary Figure S8). Comparing the potency of single domains to full-length protein, it became obvious that, e.g., A 410099.1 gained about 10-fold in potency binding to the single BIR3 domain compared to the full-length protein whereas for AZD5582 a larger increase in affinity was observed (Supplementary Figure S7).

## Discussion

In this manuscript, we have established a family-wide screening panel for BIR domain proteins. The phylogenetic analysis of the BIR domains indicated not only the classical three BIR domain groups (BIR1-BIR3) but also an ungrouped BIRC1-BIR[2] and a fourth “BIR4” domain group (BIRC1-BIR[1], BIRC5, BIRC6), the functional importance of which still needs to be determined (Figure 1C). Both, BIRC5 and BIRC6 have been already shown to have alterations in their BIR domain sequences leading to different cellular functions than the ones shown for the E3-ligases (Cao et al., 2008). However, the BIR domains of BIRC1 clustered within the BIR1 domain family (third BIR domain, BIRC1-BIR1), BIR4 domain (BIRC1-BIR[1]) and an intermediate branch rooting between BIR1 and BIR3 domains (Figure 1). This diverse distribution across the BIR domain family tree can be explained by genomic analysis of chromosome 5 which suggests that an inverted chromosome duplication is the reason for the genetic location of BIRC1 including the proximity to *SMA*. Alterations in this 1–2 Mb duplication are directly linked to spinal muscular atrophy (SMA) and BIRC1 has been suggested to have an SMA modulating role (Schmutz et al., 2004; Maier et al., 2007). However, this inverted duplication together with sequence alteration may have led to the degeneration of the BIR domains from their initial type 1 and type 2 sequences.

The E3 BIRCs, BIRC2-4, together with BIRC1-BIR1 encode a BIR1 domain. The BIR1 domains of BIRC2 and BIRC3 do not possess the IBM grove found in BIR3 and BIR2 domains, but instead interact with TNF Receptor-Associated Factor 2 (TRAF2) as part of the TNFα signaling pathway (Rothe et al., 1995). This pathway activates the RIPK-dependent apoptosis through caspase 3 and necroptosis (Garrison et al., 2009). Likewise the BIR2 domain of BIRC4 binds to the kinase domain of RIPK2 activating NF-kB and cytokine signaling (Goncharov et al., 2018; Hrdinka et al., 2018). Also the BIR1 domain of BIRC4, has been shown to play a role during the NF-kB activation via its interaction with TAB1 (Lu et al., 2007). The presence of a BIR1 domain in BIRC1 (BIRC1-BIR1) therefore suggests involvement in these pathways as well. Additionally, the BIR2 domains bind SMAC/DIABLO and caspase 3 suggesting involvement in the anti-apoptotic effects together with BIR3 domains, which bind SMAC/DIABLO and caspase 9 for proteasomal degradation (Verhagen et al., 2001).

As the published affinity data was mainly obtained from the BIRC2-BIR3 domain (Table 1), we chose the same domain for the *in vitro* affinity validation. Data obtained by the fluorescence polarization assay showed low nanomolar *K*_I_ values for the different SMAC mimetics, which generally agreed with published data. In our assay, most of the compound titrations showed 2 data points (2 and 5 nM) with significantly higher mP values with respect to the residual data points, indicating, that the ‘assay wall’ is located between the used peptide tracer concentration and the peptide tracers *K*_D_. Since the majority of calculated *K*_I_ values were smaller than 5 nM, and therefore smaller than the detection limit, the general conclusion for of the FP assay is that all SMAC mimetics have shown extraordinary high affinities of smaller than 10 nM towards BIRC2-BIR3 proving the FP assay to be applicable for interaction determination of compounds with lower affinity while the non-SMAC mimetic UC-112 has shown no interaction at all. UC-112 has been reported as an inhibitor for BIRC5 (Survivin) based on molecular modelling and cellular data monitoring apoptosis (Wang et al., 2018). The docking model for UC-112 into BIRC5 has shown interaction to the putative IBM groove which was confirmed by a crystal structure of BIRC5 with the SMAC peptide bound (pdb: 3UIH). Therefore, the binding into the same grove in BIRC2-BIR3 was expected at least with low affinity. Our analysis of the binding affinity of our test compound set to the full-length and single BIR domains of BIRCs revealed UC-112, a putative BIRC5 inhibitor to be inactive in any of the cellular as well as biophysical assays.

Due to the small hydrophobic core of BIR3 domains and the high melting temperature of ∼74°C, the peak height during the DSF measurements were low due to the temperature dependence of fluorescence. However, DSF provided a fast readout with significant thermal shifts in melting temperatures. Since the compound was provided in excess over the protein, no improved melting temperatures for possible protein-protein interfaces were expected for the bivalent compounds due to the occupancy of the binding sites by individual inhibitors instead of the bivalent binding mode. AZD5582 showed higher melting temperatures in comparison to not only the other bivalent compound but also compared to the monovalent ligands. We did not observe for every compound a good correlation between the *in vitro* assays, FP and DSF. However, exact correlation between the two assay formats is not to be expected since the assay principles differ significantly. While DSF requires the protein binding sites to be saturated with compound, FP assay uses dose-response measurements. Nevertheless, the excess of ligand used in DSF may lead to concentrations near the solubility limit of the used ligands.

For all tested BIRCs the tested tracer was slightly more potent on the full-length protein compared to the single BIR domains in the cellular NanoBRET assay. The difference of the truncated proteins towards their full-length parent constructs can originate from differences in e.g., post-translational modifications, higher oligomeric states or localization of the protein. Possible reasons for this could be the truncation of regions responsible for post-translation modification attachment, oligomerization or import sequences into different cell compartments.

The bivalent compounds BV-6 and Birinapant had surprisingly low potency in cell-based NanoBRET assays compared to the biophysical characterization. Since it has been shown that both compounds had nanomolar affinity towards BIRCs in the FP assay and ITC, their cell permeability might be the reason for the considerable drop in cellular potency. Nevertheless, the bivalent SMAC mimetics (SM-164 and AZD5582) were found to be well cell membrane permeable as indicated by their comparable affinity to the monovalent SMAC mimetics. However, biological activities such as cell viability, degradation of c-IAP1 and activation of noncanonical NF-kB pathway may well differ from the measured on-target effect for individual BIRCs, in particular given the promiscuous nature of the compounds*.*

Interestingly, two compounds were identified that showed preferential binding to BIRC7. AZD5582 is more than 10-fold more potent for full-length BIRC7 compared with any of the remaining full-length proteins (Table 2). This shift in potency was only observed in full-length constructs while the single BIR3 domains showed comparable potencies (Table 3). Since BIRC7 does not have multiple BIR domains, the bivalent binding to two intrinsic BIR domains cannot be an explanation towards its increased potency for this inhibitor. In the full-length proteins, binding seems to be influenced through additional domain structures or complex formation. BIRC2 and BIRC3 have been reported to assume an active as well as an inactive state in which the IBM groove is blocked by an interaction with the RING domain, creating a closed state of the protein (Dueber et al., 2011). Therefore, compound interactions for which higher affinities towards the single domain were observed could be due to the absence of auto-inhibition in the truncated constructs. Dueber et al. however, also revealed that BIRC antagonists (SMAC mimetics) can induce the active conformation and binding is therefore not always hindered by different conformational states and domain interaction in the context of the protein.

Future structural studies on the single BIRC7-BIR domain bound to AZD5582 and CUDC-427 may provide insights for the development of BIRC7 selective inhibitors. We also observed some additional domain selectivity: AZD5582 and A 410099.1 showed significantly higher affinity towards BIR2 domains compared to other inhibitors. Some of the investigated compounds, such as AT406, SM-164 and AZD5582 bound to the BIRC1-BIR[2] domain, which is an atypical BIR domain that has not prior been shown to interact with SMAC mimetics, expanding the target scope of SMAC mimetics outside the E3 ligases family members.

## Conclusion

In this work, a BIRC family-wide selectivity screening platform was established. This selectivity toolbox comprises all full-length E3 ligases, the non-E3 ligase BIRC5 and 7 of the 14 single BIR domains of this family. The here presented selectivity panel therefore consists of BIRC1 (BIR[2]), BIRC2 (FL, BIR2 and BIR3), BIRC3 (FL, BIR2 and BIR3), BIRC4 (FL), BIRC5 (FL), BIRC7 (FL and BIR) and BIRC8 (FL and BIR). Hence, all five E3 ligases can now be used for selectivity profiling of putative inhibitors in living cells. Due to the similarities of their BIR3 domains, selectivity of the BIRC E3 ligases may be difficult to achieve. This setting allows to evaluate selectivity profiles of E3 ligands, novel chemical probes and PROTACs with the possibility of domain specific screening campaigns.

Strikingly, most of the tested compounds did not show any selectivity within the BIRC family, including clinical candidates like LCL161, Birinapant and AT406 (Xevinapant) defining them as pan-BIRC inhibitors. Due to the general involvement of BIRC proteins in a diverse range of pathways, a pan-BIRC inhibitor could lead to unwanted effects which could be avoided by a development of selective BIRC inhibitors targeting only the BIRC proteins involved in the specific signalling pathway. Since BIRC1 has been shown to play a role in response to microbial infections (Vance, 2015), the inhibitors AZD5582, AT406 and SM-164, which showed high affinity for BIRC1, could be studied as anti-infective agents. AZD5582 and CUDC-427 showed moderate selectivity within the tested full-length BIRC family favouring BIRC7 (4−10-fold). This property positions these scaffolds as potential chemical starting points towards selective BIRC7 inhibitors, while BIRC family-wide screening campaigns can potentially yield more candidates for a more diverse range of BIRC family members. The here established cellular toolbox will prove useful in the development of superior and more selective BIRC inhibitors, chemical probes and for the optimization of future clinical candidates.

## Data Availability

The original contributions presented in the study are included in the article/Supplementary Material, further inquiries can be directed to the corresponding authors.
