# Supplementary material for: A Toolbox for the Generation of Chemical Probes for Baculovirus IAP Repeat Containing Proteins
Source: Front Cell Dev Biol. 2022 May 26;10:886537. doi: 10.3389/fcell.2022.886537 (PMC9204419; doi:10.3389/fcell.2022.886537)
Supplement: Supplementary file 1 [file DataSheet1.pdf]

A toolbox for the generation of chemical probes for BIR domain containing proteins

Martin P. Schwalm[1,2†], Lena M. Berger[1,2†], Maximilian Meuter[1], James D. Vasta[3], Cesear R. Corona[3], Sandra Röhm[1,2], Benedict-Tilman Berger[1,2], Frederic Farges[1], Sebastian Beinert[1], Franziska Preuss[1,2], Viktoria Morasch[1,2], Vladimir V. Rogov[1,2], Sebastian Mathea[1,2], Krishna Saxena[1,2], Matthew B. Robers[3], Susanne Müller[1,2*], Stefan Knapp[1,2,4*]

[1]Institute for Pharmaceutical Chemistry, Department of Biochemistry, Chemistry and Pharmacy, Goethe University, 60438 Frankfurt, Germany

[2]Structural Genomics Consortium, Buchmann Institute for Molecular Life Sciences, Goethe University, 60438 Frankfurt, Germany

[3]Promega Corporation, 2800 Woods Hollow Road, Madison, WI 53719, USA

[4]German Cancer Consortium (DKTK) / German Cancer Research Center (DKFZ), DKTK site Frankfurt-Mainz, 69120 Heidelberg, Germany

Table of contents

Supplementary Figure S1: Chemical structures of the literature compounds used in this study

Supplementary Figure S2. Multiple sequence alignment of the different BIR domains

Supplementary Figure S3: Results of the fluorescence polarization (FP) assay

Supplementary Figure S4: Melting temperature curves

Supplementary Figure S5: Results of the isothermal titration calorimetry (ITC) experiments

Supplementary Figure S6: Tracer titration results for the tested BIRC constructs

Supplementary Figure S7: Compound titration results for the investigated BIRC constructs

Supplementary Figure S8: Illustration of the SMAC mimetic selectivity and the IAP Tracer (Promega) towards in cellular target engagement assay tested constructs.

Supplementary Figure S9: NanoBRET data reliability represented by individual replicates of selected compound-protein pairs.

Supplementary Figure S10: MS of tracer molecule

Supplementary Figure S11: Compound quality

Supplementary Table S1: DSF results

Supplementary Table S2: Boundaries used, tracer concentration, and overall assay quality of NanoBRET constructs.

Supplementary Material

1.1 Supplementary Figures and Tables

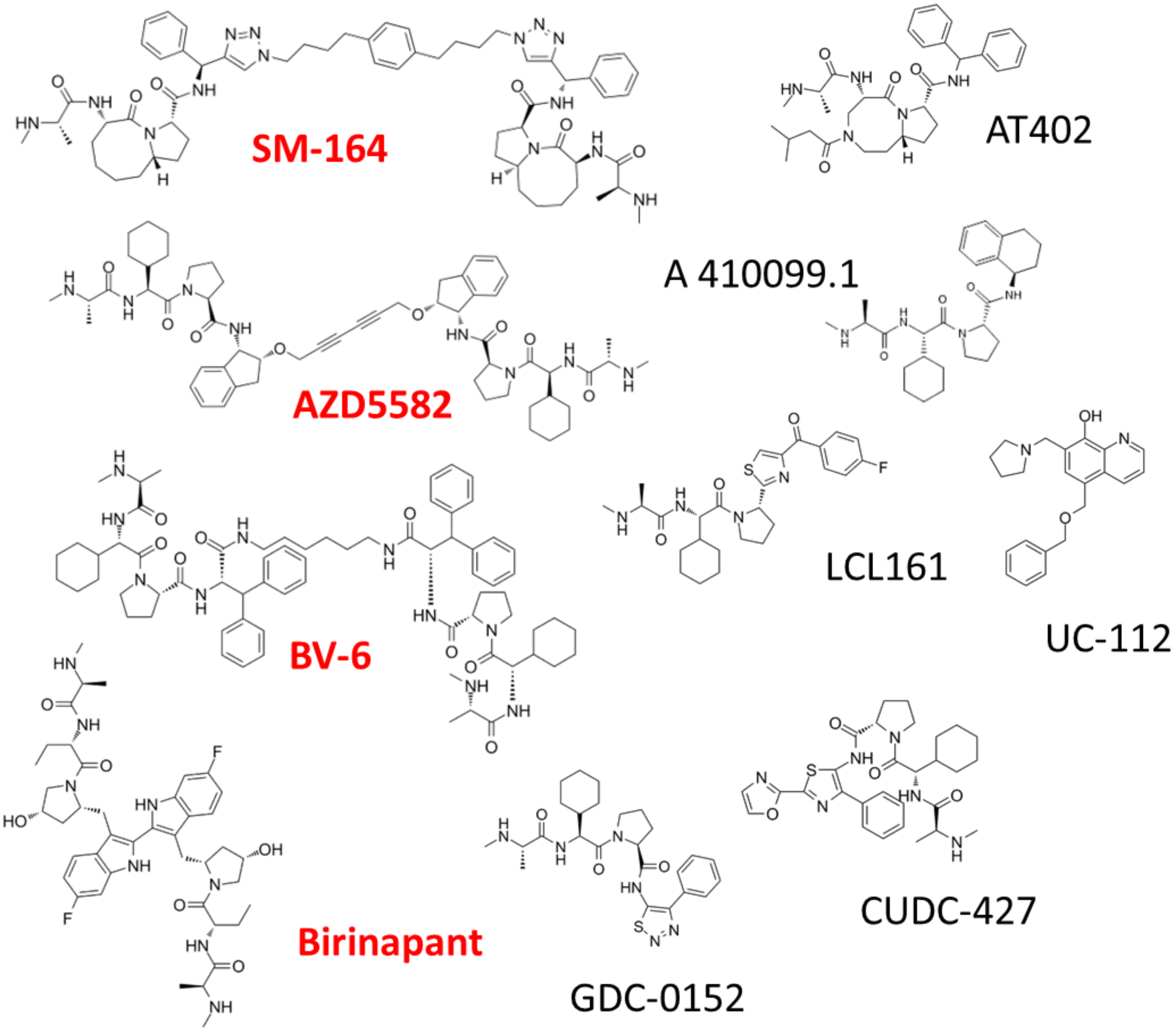

Supplementary Figure S1. Chemical structures of the literature compounds used in this study. Bivalent compounds are marked red.

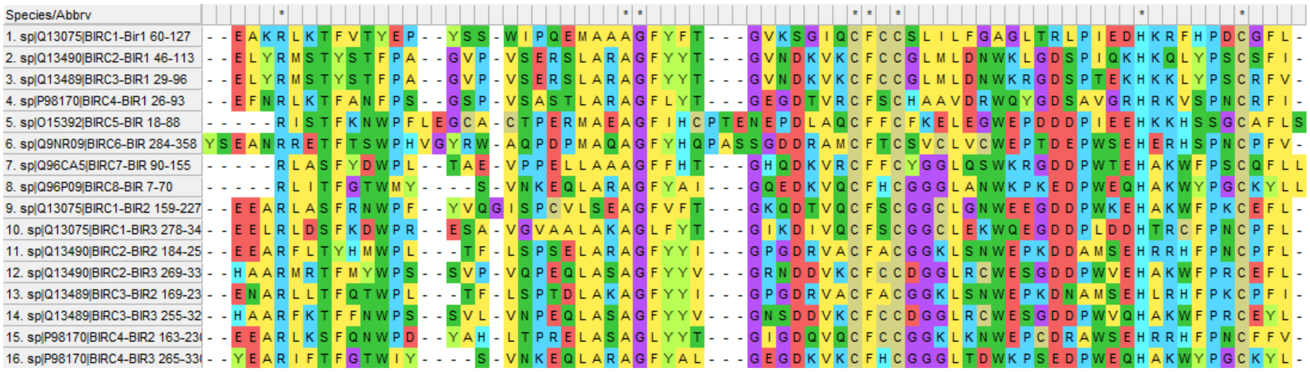

Supplementary Figure S2. Multiple sequence alignment of the different BIR domains depicted with UniProt ID, BIRC family member and respective BIR domain boundaries.

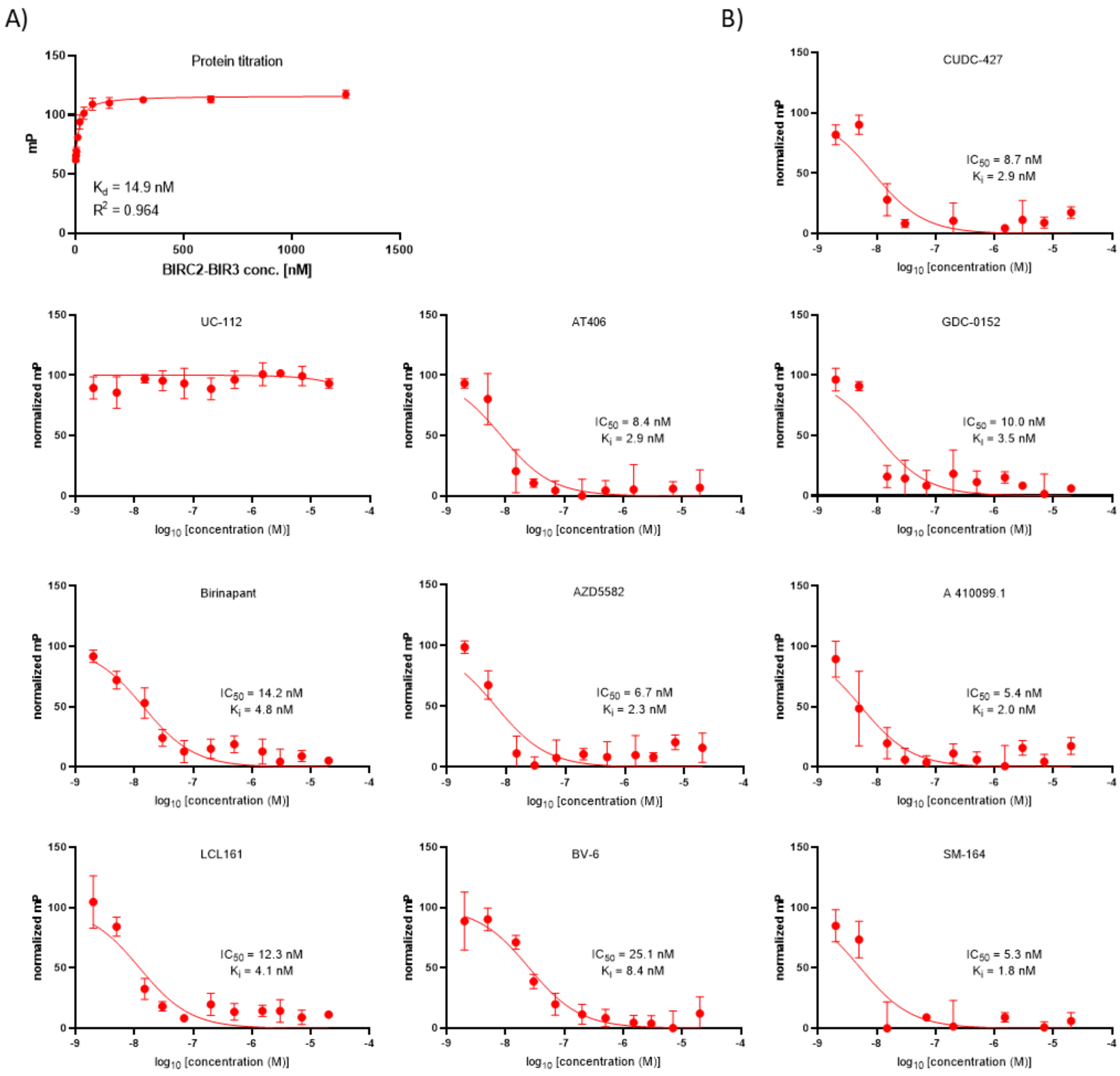

Supplementary Figure S3. Results of the fluorescence polarization (FP) assay. A) protein titration to 5 nM of the tracer peptide with the resulting fit and K_D of the peptide to the BIRC2-BIR3 domain. B) compound titration curves with IC_{50} values and calculated K_I values. Compound names are depicted at the top of each frame. Data were expressed as mean ± SD (n=3).

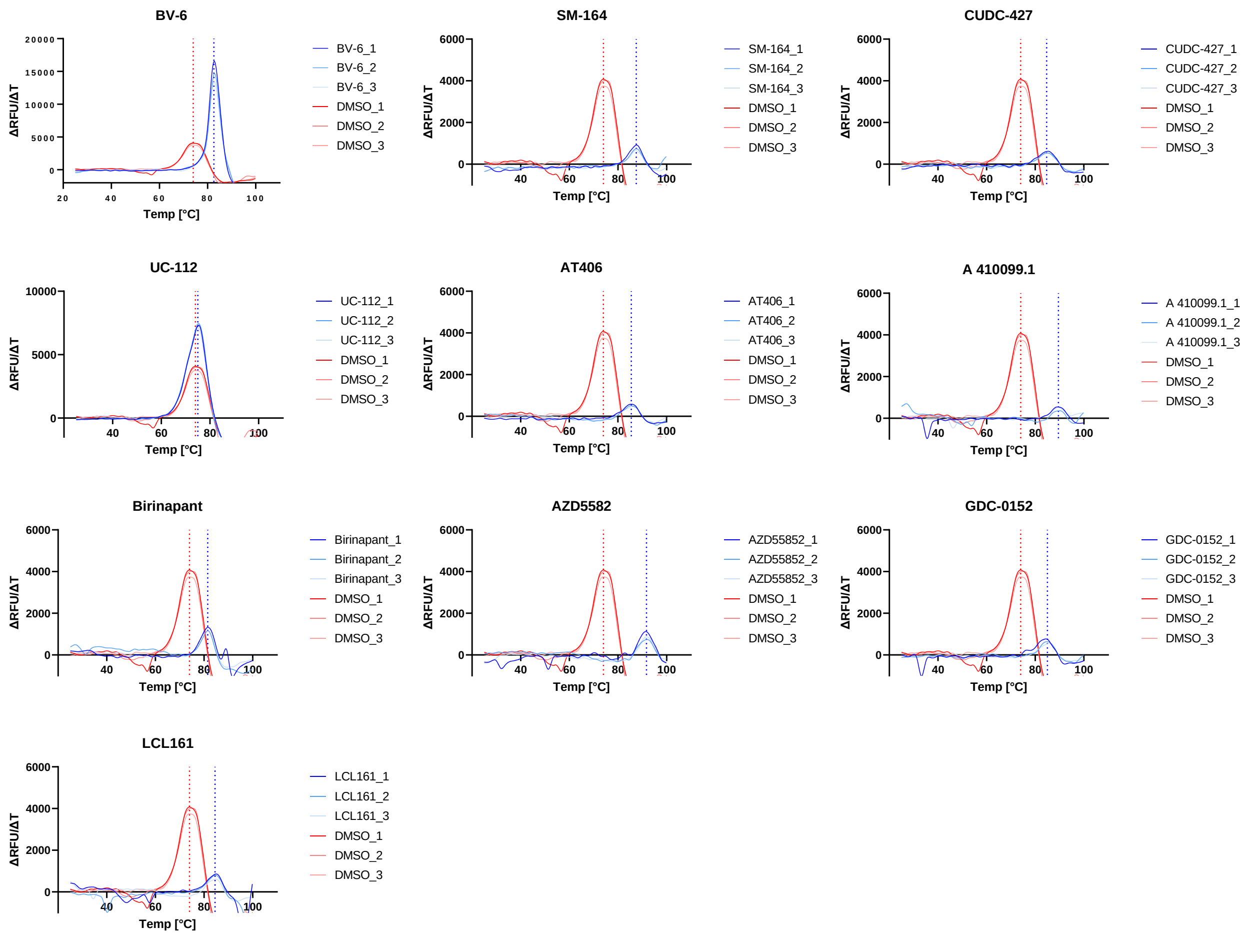

Supplementary Figure S4. Melting temperature curves (ΔRFU/ΔT) of the compound treated BIR3 domain of BIRC2 (blue) in comparison to the DMSO control curves (red). Each frame shows the measurements in triplicates with dotted lines indicating the melting temperature for the control (red) and the compound (blue) (n=3).

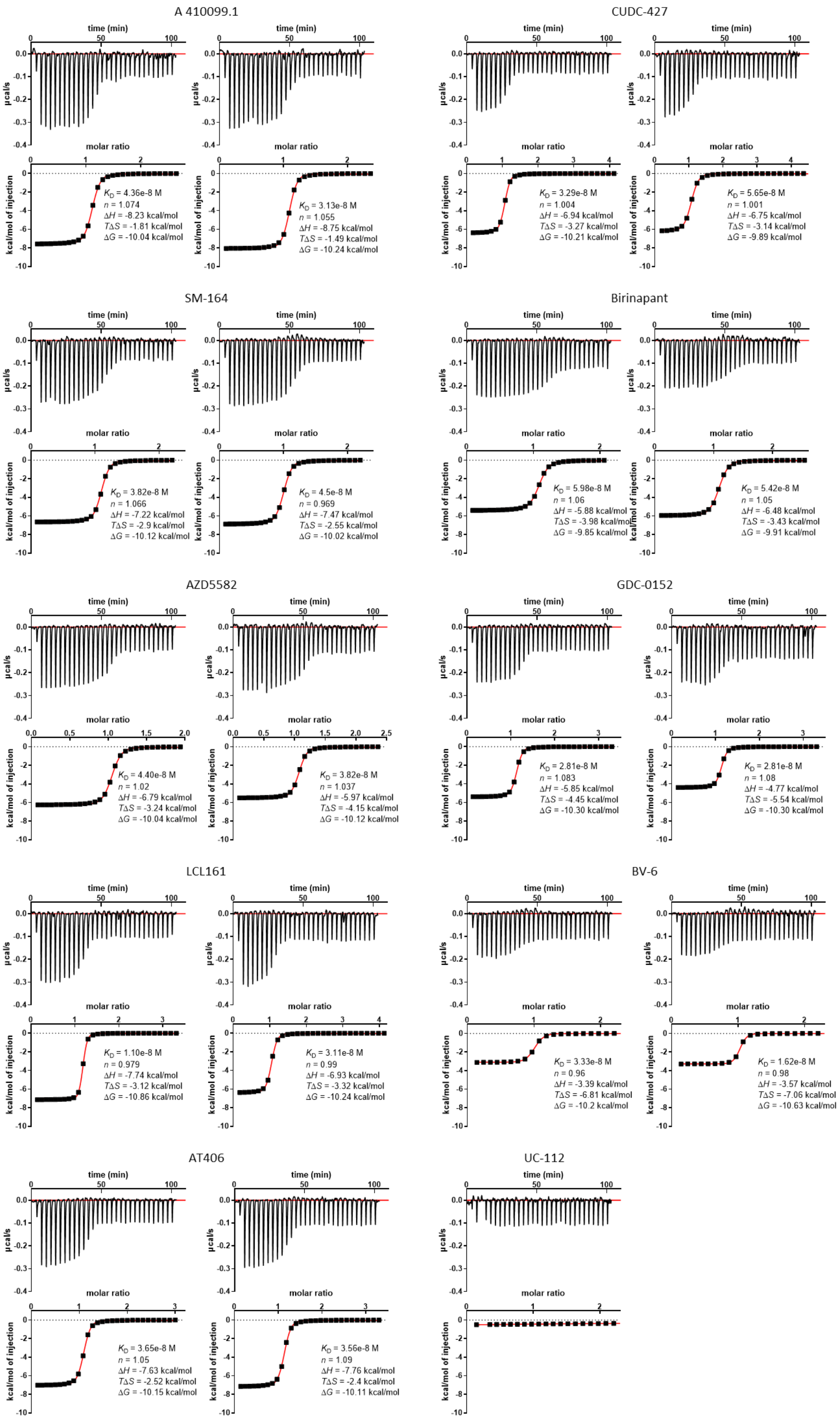
A 410099.1
K_D = 4.36e-8 M
n = 1.074
ΔH = -8.23 kcal/mol
$T\Delta S$ = -1.81 kcal/mol
ΔG = -10.04 kcal/mol
K_D = 3.13e-8 M
n = 1.055
ΔH = -8.75 kcal/mol
$T\Delta S$ = -1.49 kcal/mol
ΔG = -10.24 kcal/mol
CUDC-427
K_D = 3.29e-8 M
n = 1.004
ΔH = -6.94 kcal/mol
$T\Delta S$ = -3.27 kcal/mol
ΔG = -10.21 kcal/mol
K_D = 5.65e-8 M
n = 1.001
ΔH = -6.75 kcal/mol
$T\Delta S$ = -3.14 kcal/mol
ΔG = -9.89 kcal/mol
SM-164
K_D = 3.82e-8 M
n = 1.066
ΔH = -7.22 kcal/mol
$T\Delta S$ = -2.9 kcal/mol
ΔG = -10.12 kcal/mol
K_D = 4.5e-8 M
n = 0.969
ΔH = -7.47 kcal/mol
$T\Delta S$ = -2.55 kcal/mol
ΔG = -10.02 kcal/mol
Birinapant
K_D = 5.98e-8 M
n = 1.06
ΔH = -5.88 kcal/mol
$T\Delta S$ = -3.98 kcal/mol
ΔG = -9.85 kcal/mol
K_D = 5.42e-8 M
n = 1.05
ΔH = -6.48 kcal/mol
$T\Delta S$ = -3.43 kcal/mol
ΔG = -9.91 kcal/mol
AZD5582
K_D = 4.40e-8 M
n = 1.02
ΔH = -6.79 kcal/mol
$T\Delta S$ = -3.24 kcal/mol
ΔG = -10.04 kcal/mol
K_D = 3.82e-8 M
n = 1.037
ΔH = -5.97 kcal/mol
$T\Delta S$ = -4.15 kcal/mol
ΔG = -10.12 kcal/mol
GDC-0152
K_D = 2.81e-8 M
n = 1.083
ΔH = -5.85 kcal/mol
$T\Delta S$ = -4.45 kcal/mol
ΔG = -10.30 kcal/mol
K_D = 2.81e-8 M
n = 1.08
ΔH = -4.77 kcal/mol
$T\Delta S$ = -5.54 kcal/mol
ΔG = -10.30 kcal/mol
LCL161
K_D = 1.10e-8 M
n = 0.979
ΔH = -7.74 kcal/mol
$T\Delta S$ = -3.12 kcal/mol
ΔG = -10.86 kcal/mol
K_D = 3.11e-8 M
n = 0.99
ΔH = -6.93 kcal/mol
$T\Delta S$ = -3.32 kcal/mol
ΔG = -10.24 kcal/mol
BV-6
K_D = 3.33e-8 M
n = 0.96
ΔH = -3.39 kcal/mol
$T\Delta S$ = -6.81 kcal/mol
ΔG = -10.2 kcal/mol
K_D = 1.62e-8 M
n = 0.98
ΔH = -3.57 kcal/mol
$T\Delta S$ = -7.06 kcal/mol
ΔG = -10.63 kcal/mol
AT406
K_D = 3.65e-8 M
n = 1.05
ΔH = -7.63 kcal/mol
$T\Delta S$ = -2.52 kcal/mol
ΔG = -10.15 kcal/mol
K_D = 3.56e-8 M
n = 1.09
ΔH = -7.76 kcal/mol
$T\Delta S$ = -2.4 kcal/mol
ΔG = -10.11 kcal/mol
UC-112
time (min)
µcal/s
molar ratio
kcal/mol of injection

Supplementary Figure S5. Results of the isothermal titration calorimetry (ITC) experiments. Each frame shows the curve obtained from the measurement with the baseline subtracted (top) and the curve plotted from the integrals of each binding peak to calculate the K_D of each compound (bottom). Duplicates were measured for each compound except for UC-112 which has shown no binding in agreement with the other biophysical assays (n=2).

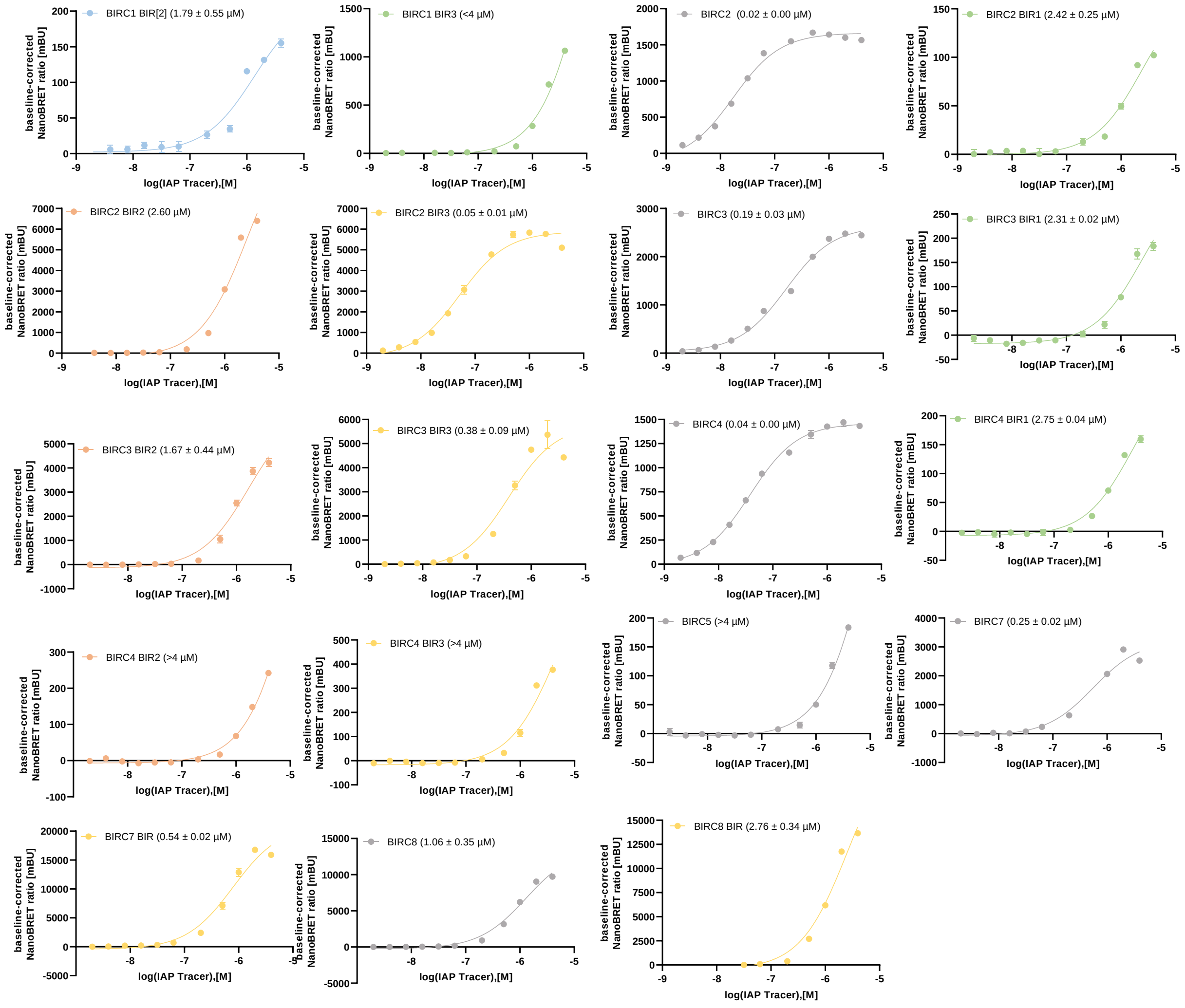

Supplementary Figure S6. Tracer titration results for the tested BIRC constructs. The baseline-corrected NanoBRET ratio (610nm/450nm) is plotted against the log concentration of titrated Tracer. Data were expressed as mean ± SEM using two independent experiments performed in duplicates. The colors of the curves correspond with the colors used in Figure 1 C. BIR1 (green), BIR2 (orange), BIR3 (yellow), full-length constructs (grey) and BIR4 domains (blue). The Tracer $K_{D,app}$ is displayed in the upper left corner of each graph (n=4).

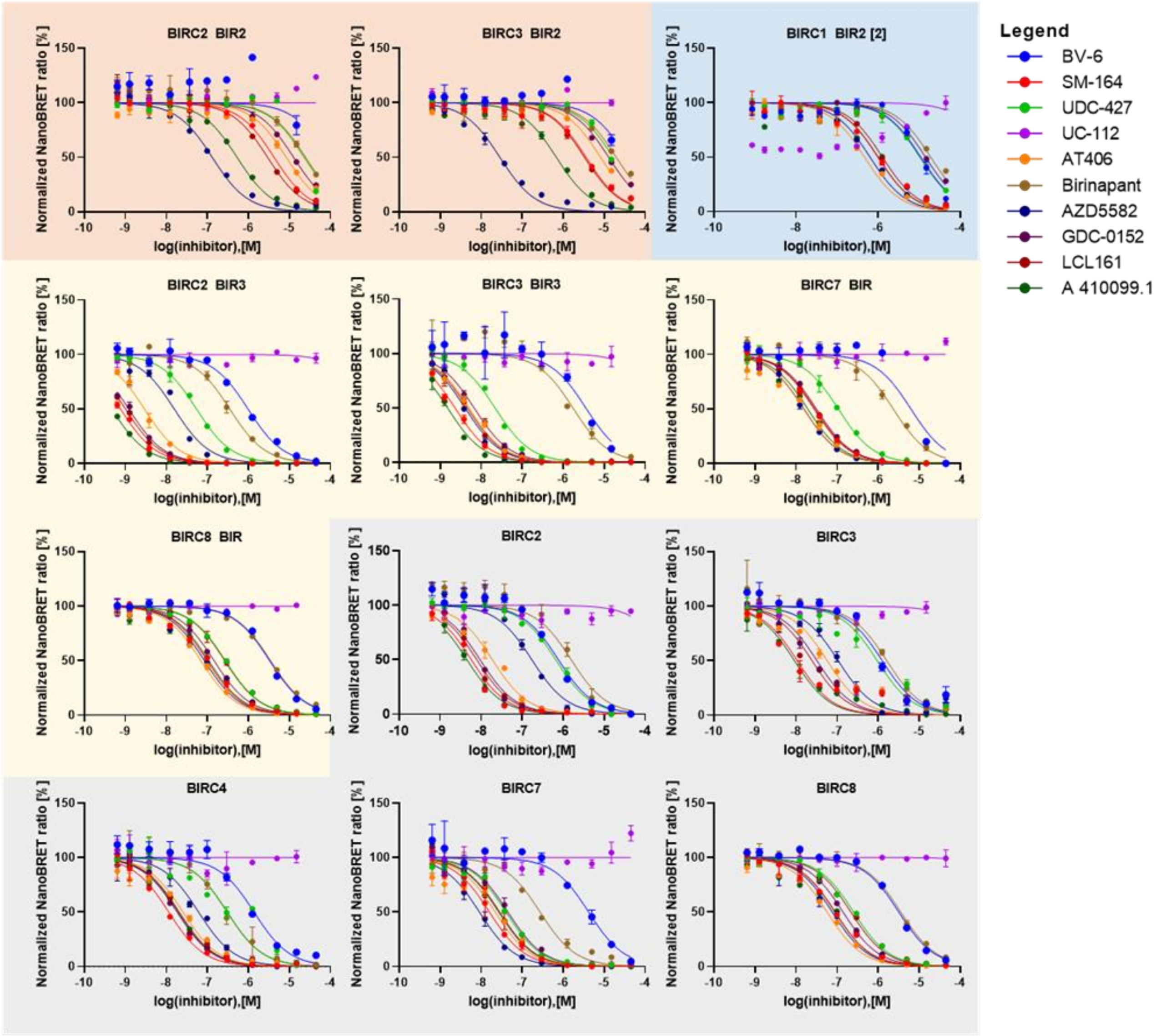

Supplementary Figure S7. Compound titration results for the investigated BIRC constructs. Data were expressed as mean ± SEM using two independent experiments performed in duplicates. The background colors of the curves correspond with the colors used in Figure 1 C. BIR1 (green), BIR2 (orange), BIR3 (yellow), full-length constructs (grey) and BIR4 domains (blue). The related IC_{50} can be found in Table 3 (full-length constructs) and Table 4 (single BIR domains) (n=4).

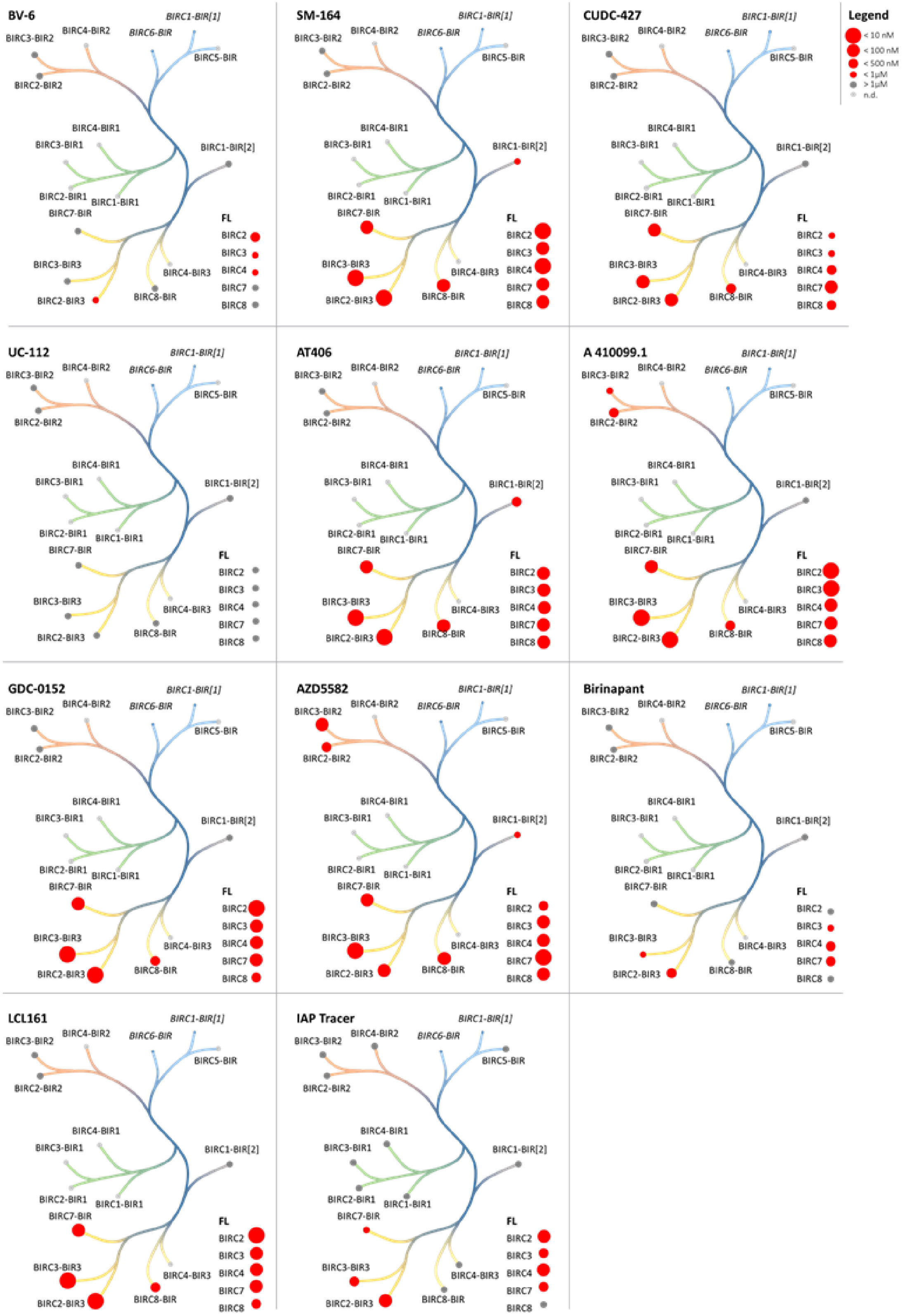

Supplementary Figure S8. Illustration of the SMAC mimetic selectivity and the IAP Tracer (Promega) towards in cellular target engagement assay tested constructs. Non-working constructs are written in italic.

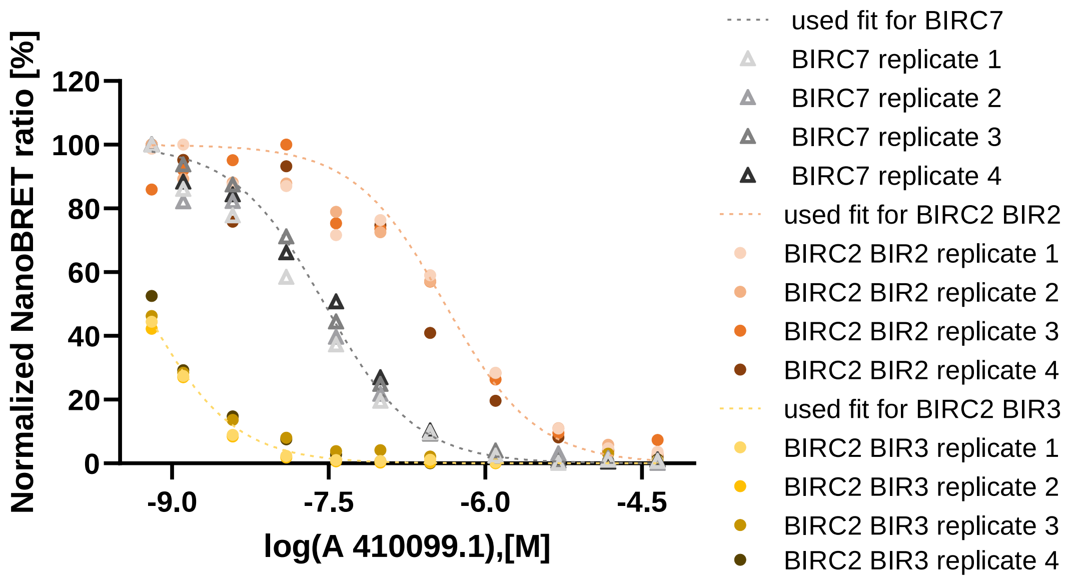

Supplementary Figure S9. A 410099.1 titration curves of individual replicates of BIRC7, BIRC2 BIR2 and BIRC2 BIR3. The dashed line shows the corresponding fit calculated for the mean of each experiment. The data was collected as two individual experiments performed in duplicates (n=4).

Supplementary Figure S10. ASL Chromatogram and % purity report of tracer molecule **(**separate file)

Supplementary Figure S11. Quality control of used compounds (separate file)

Supplementary Table S1: DSF results are specified as the difference in melting temperature (°C) to the DMSO control (n=3).

Compound	ΔT_m [°C]			Mean, ΔT_m [°C]	±, [°C]
BV-6	9.1	9.1	9.1	9.1	0.0
SM-164	13.6	13.4	13.1	13.4	0.2
CUDC-427	10.9	10.9	10.8	10.9	0.1
UC-112	0.4	0.3	0.2	0.3	0.1
AT406	11.8	11.8	11.4	11.6	0.2
Birinapant	7.5	7.3	6.8	7.2	0.3
AZD5582	18.2	18.0	17.9	18.0	0.1
GDC-0152	11.1	11.0	9.6	10.6	0.7
LCL161	10.5	10.2	9.9	10.2	0.3
A 410099.1	15.8	15.7	15.6	15.7	0.1

Supplementary Table S2: Boundaries used, tracer concentration, and overall assay quality of NanoBRET constructs. The overall assay quality is stated in the last column. Data for tracer $K_{d,app}$ were expressed as mean ± SEM using two independent experiments performed in duplicates. A good assay is determined by an average luciferase signal higher than 1000, a z' higher than 0.5 and an assay window higher than 2.0. The assay window describes the dynamic range of an assay and is the ratio between the highest signal and the lowest signal (100% control/ 0% control). If these criteria are not matched, the assay quality is considered "low" and the compound titrations are not shown. (n=4)

Construct	Vector-Construct	N-terminus	C-terminus	Tracer $K_{d,app}$ [µM]	Tracer used [µM]	Average Luciferase Signal	z'	Assay window	Overall Assay Quality
BIRC1 BIR[1]	pF-31Kp-BIRC1-BIR[1]	G28	R148	n.d.	1	9733	-0.9	1.3	low
BIRC1 BIR[2]	pF-31Kp-BIRC1-BIR[2]	D139	G251	1.79 ± 0.55	1	8045	0.74	18.2	high
BIRC1 BIR1	pF-31Kp-BIRC1-BIR1	N256	Q391	> 4.00	1	10705.7	-0.1	2.3	low
BIRC2	Promega (pF-31Kp)	M1	S618	0.02 ± 0.00	0.05	28688.9	0.6	7	high
BIRC2 BIR1	pF-31Kp-BIRC2-BIR1	D24	S134	2.42 ± 0.25	1	10068.3	-1	1.3	low
BIRC2 BIR2	pF-31Kp-BIRC2-BIR2	A165	Q267	2.6	1	14900	0.7	22.8	high
BIRC2 BIR3	pF-31Kp-BIRC2-BIR3	E251	D372	0.05 ± 0.01	0.05	11261.1	0.9	20.6	high
BIRC3	Promega (pF-31Kp)	M1	S604	0.19 ± 0.03	0.2	20545.3	0.7	8	high
BIRC3 BIR1	pF-31Kp-BIRC3-BIR1	N6	S116	2.31 ± 0.02	1	5651.7	-0.1	1.7	low
BIRC3 BIR2	pF-31Kp-BIRC3-BIR2	N147	Q253	1.67 ± 0.44	1	10649.8	0.8	18.1	high
BIRC3 BIR3	pF-31Kp-BIRC3-BIR3	E236	S360	0.38 ± 0.09	0.4	4769.2	0.7	22.6	high
BIRC4	Promega (pF-31Kp)	M1	S301	0.04 ± 0.00	0.05	11924.1	0.6	4.3	high
BIRC4 BIR1	pF-31Kp-BIRC4-BIR1	N4	Q114	2.75 ± 0.04	1	6500.4	-0.8	1.5	low
BIRC4 BIR2	pF-31Kp-BIRC4-BIR2	G144	S253	> 4.00	1	10189.8	-0.5	1.4	low
BIRC4 BIR3	pF-31Kp-BIRC4-BIR3	S245	N373	> 4.00	1	3613.8	-0.9	1.5	low
BIRC5	pF-31Kp-BIRC5	M1	D142	> 4.00	1	11998.8	0.2	1.4	low
BIRC7	pF-31Kp-BIRC7	M1	S298	0.25 ± 0.02	0.25	6963	0.7	10.8	high
BIRC7 BIR	pF-31Kp-BIRC7-BIR	G70	S176	0.54 ± 0.02	0.55	5391.5	0.8	73.2	high
BIRC8	pF-31Kp-BIRC8	M1	S236	1.06 ± 0.35	1	4977.5	0.8	42.9	high
BIRC8 BIR	pF-31Kp-BIRC8-BIR	M1	A90	2.76 ± 0.34	1	5224.4	0.9	78.5	high
